# Congenital diaphragmatic hernia outcomes: navigating center-to-center variability in level 4 NICUs in the Children’s Hospitals Neonatal Consortium

**DOI:** 10.1038/s41390-025-03829-0

**Published:** 2025-02-25

**Authors:** Yigit S. Guner, Isabella Zaniletti, Theresa R. Grover, Sharada H. Gowda, Nicolas F. M. Porta, Michael A. Padula, Sarah Keene, Burhan Mahmood, Jacquelyn R. Evans, Holly L. Hedrick, Karna Murthy

**Affiliations:** 1 Department of Surgery, University of California Irvine and Children’s Hospital of Orange County, Orange, CA USA; 2 Children’s Hospitals Neonatal Consortium, Dover, DE USA; 3 Children’s Hospital Colorado, University of Colorado School of Medicine, Aurora, CO USA; 4 Baylor College of Medicine, Texas Children’s Hospital, Houston, TX USA; 5 Ann & Robert H. Lurie Children’s Hospital of Chicago, Northwestern University, Chicago, IL USA; 6 Children’s Hospital of Philadelphia and University of Pennsylvania Perelman School of Medicine, Philadelphia, PA USA; 7 Children’s Healthcare of Atlanta and Emory University School of Medicine, Atlanta, GA USA; 8 University of Pittsburgh School of Medicine, Pittsburgh, PA USA; 9 Children’s Healthcare of Atlanta—Egleston, Atlanta, GA USA; 10 Children’s Healthcare of Atlanta—Scottish Rite, Atlanta, GA USA; 11 Dell Children’s, Austin, TX USA; 12 Children’s of Alabama, Birmingham, AL USA; 13 Boston Children’s Hospital, Boston, MA USA; 14 Levine Children’s Hospital, Charlotte, NC USA; 15 Ann & Robert H. Lurie Children’s Hospital, Chicago, IL USA; 16 Cincinnati Children’s Hospital, Cincinnati, OH USA; 17 Cleveland Clinic, Cleveland, OH USA; 18 Nationwide Children’s Hospital, Columbus, OH USA; 19 Children’s Medical Center, Dallas, TX USA; 20 Children’s Hospital Colorado, Denver, CO USA; 21 Children’s Hospital Michigan, Detroit, MI USA; 22 Cook Children’s Healthcare System, Ft. Worth, TX USA; 23 Connecticut Children’s, Hartford, CT USA; 24 Texas Children’s Hospital, Houston, TX USA; 25 Riley Children’s Health, Indianapolis, IN USA; 26 University of Iowa Stead Family Children’s Hospital, Iowa City, IA USA; 27 The Children’s Mercy Hospital, Kansas City, MO USA; 28 Arkansas Children’s, Little Rock, AR USA; 29 Los Angeles Children’s Hospital, Los Angeles, CA USA; 30 American Family Children’s Hospital, Madison, WI USA; 31 Le Bonheur Children’s Hospital, Memphis, TN USA; 32 Children’s Minnesota, Minneapolis, MN USA; 33 Wisconsin Children’s Hospital, Milwaukee, WI USA; 34 UCSF Benioff Children’s Hospital, Oakland, CA USA; 35 Oklahoma Children’s Hospital, Oklahoma, OK USA; 36 Omaha Children’s Hospital, Omaha, NE USA; 37 Children’s Hospital of Orange County, Orange, CA USA; 38 AdventHealth for Children, Orlando, FL USA; 39 Nemours Children’s Hospital, Orlando, FL USA; 40 Children’s Hospital of Philadelphia, Philadelphia, PA USA; 41 St. Christopher’s Hospital for Children, Philadelphia, PA USA; 42 Phoenix Children’s Hospital, Phoenix, AZ USA; 43 Pittsburgh Children’s Hospital, Pittsburgh, PA USA; 44 Mayo Clinic Children’s, Rochester, MN USA; 45 Primary Children’s Hospital, Salt Lake City, UT USA; 46 Rady Children’s Hospital, San Diego, CA USA; 47 Seattle Children’s Hospital, Seattle, WA USA; 48 St. Louis Children’s Hospital, St. Louis, MO USA; 49 Children’s Minnesota, St Paul, MN USA; 50 All Children’s Hospital, St. Petersburg, FL USA; 51 The Hospital for Sick Children, Toronto, ON Canada; 52 Children’s National Hospital, Washington, NW USA; 53 Nemours/Alfred I. duPont Hospital for Children, Wilmington, DE USA; 54 Brenner Children’s Hospital, Winston-Salem, NC USA

## Abstract

**Background:**

This study examined inter-center variation (ICV) in inpatient outcomes for infants with congenital diaphragmatic hernia (CDH), aiming to contribute to quality metrics and clinical benchmarks in neonatal care.

**Methods:**

We retrospectively analyzed CDH cases from the Children’s Hospitals Neonatal Consortium (CHNC) database (2010–2022), focusing on infants without prior surgical repair or discharge. Our outcomes of interest included inpatient survival, survival without ECMO, and hospital length of stay (LOS). We incorporated centers with ≥30 cases into multivariable models to adjust for patient and clinical factors.

**Results:**

Analysis of 3639 infants revealed significant ICV. Unadjusted inpatient survival rate was 76.5%, with ICV ranging from 55.4% to 90.7%. The composite outcome of survival without ECMO was 63.3% (ICV: 38.6–87.9%). The median LOS for survivors was 50 days (ICV: 29–68 days). Multivariable analyses confirmed these trends, indicating an 18-fold variation in survival, a 35-fold variation in survival without ECMO, and a 3.3-fold variation in LOS across centers (*p* < 0.001 for all).

**Conclusion:**

The treating center was a significant predictor of risk-adjusted inpatient outcomes for infants with CDH. These findings highlight substantial disparities in care and support the integration of these metrics into future research and quality improvement efforts in level IV NICUs.

**Impact statement:**

- This study reveals considerable inter-center differences in CDH outcomes, contributing extensive, multicenter data to the existing body of literature. It underscores how center-specific practices affect survival and ECMO use, suggesting that organized high-level care could enhance outcomes for CDH patients. These insights lay the groundwork for center-specific quality improvement initiatives to elevate the standard of care.

## Introduction

Congenital diaphragmatic hernia (CDH) confers risks of inpatient mortality, morbidity, and cardiopulmonary sequelae. In recent years, survival to hospital discharge has likely increased, support with extracorporeal support (ECMO) has plateaued around ~25% of infants affected by CDH,^1,2^ and multiple reports regarding specific practices, and variations, have been described.^3–5^ For those who receive ECMO, risk-adjusted variations in mortality are evident between centers, as evidenced by significant discrepancies in standardized mortality ratios among centers,^3^ however, these studies do not incorporate the subjectivity or varied indications to receive ECMO. Thus, a growing body of evidence suggests center-level variation in survival for CDH, and that center is a potential variable–or a proxy for others–that contributes to variation in outcomes.

Utilizing the Children’s Hospitals Neonatal Consortium’s (CHNC) database, our group attempts to bridge the knowledge gap regarding outcomes for all infants with CDH regardless of their exposure to ECMO, and specifically, to quantify how the center of care may be associated with inpatient outcomes. CHNC is a consortium of level IV neonatal intensive care units (NICUs) collaborating to submit patient-level data on each admitted infant to improve the knowledge, safety, quality, and outcomes for these admitted children.^6^ Prior studies have shown how these data are used to quantify inter-center, inpatient outcomes for those with CDH.^7,8^ This study is novel as no prior study has demonstrated inter-center variation (ICV) inclusive of both the ECMO and non-ECMO population of CHD patients based in North America. These insights are anticipated to reveal the effects of center-specific protocols on survival rates and on the length of hospitalization for patients with CDH. We hypothesize that there exists a significant institutional-level variation in survival and length of stay outcomes for infants with CDH.

## Methods

### Data source and cohort

We conducted a secondary analysis of prospectively collected data from a cohort of infants in the Children’s Hospitals Neonatal Database (CHND). The CHND is a growing collaboration that has captured a North American cohort of infants admitted to 46 participating regional NICU’s in the US and Canada since 2010. The registry was accessed on June 30, 2023, to capture infants through 2022. Participating centers joined at different times. Using the dataset, multiple retrospective observational studies have been completed across multiple diseases and interventions, including CDH.^7–10^

### Inclusion and exclusion criteria

After identifying all infants with CDH, those who had been home prior to admission to a participating NICU were excluded as these infants were postnatally diagnosed and deemed too healthy to experience mortality. Also, those who had their surgical repair prior to admission were omitted from the analyses because of their low likelihood of being “eligible” for the outcomes of mortality or ECMO Centers with fewer than 30 (during the entire study period) were omitted to minimize the risk of imprecise estimates in generated models. Centers were omitted if infants with CDH were not primarily cared for in the participating NICUs and thus data is not captured in the CHND.

### Outcomes, exposures, and covariates

Three main outcomes were studied. The first was inpatient survival during the initial NICU stay. Infants transferred to other hospitals prior to discharge were considered survivors. Rarely, infants were hospitalized at 1 year of age, and these infants are described, but were omitted from the regression analyses. The second was the composite outcome of inpatient survival without receiving ECMO. This outcome was chosen for multiple reasons: (1) varied indications and thresholds to provide ECMO, (2) veno-arterial support is the principal form of ECMO provided for infants with CDH when offered, and (3) the known associated short-term risks (e.g., hemorrhage, thromboembolism) and consequences (e.g., carotid artery sacrifice) with veno-arterial ECMO. The third main outcome was inpatient length of stay (LOS) for the surviving infants with CDH, from date of birth to hospital discharge.

The main exposure studied was admission to a participating CHNC NICU. Other covariates included patient, clinical, and diagnostic factors that are typically known and present early in the hospital course. Though antenatal factors diagnosis of CDH was considered, specific markers of estimated fetal lung volumes were unavailable or used in this analysis because of a high proportion of missing data, variability in the timing and method of obtaining these markers clinically, and the modest predictive validity in existing studies on survival infants with CDH.

Postnatal factors considered included gestational age at birth, sex, birthweight, small for gestational age <10th centile,^11^ maternal race/ethnicity, year of birth, and Apgar scores. Disease-specific factors were the side of CDH, thoracic liver position, lowest pH in the first 12 h after admission, and the highest PCO_2_ in the first 12 h of admission_._

Associated cardiac lesions were classified into atrial septal defects (ASD), ventricular septal defect (VSD), AV septal defects, or other more complex congenital heart disease. Other diagnoses such as chromosomal and/or genetic differences, neurologic disorders, gastrointestinal anomalies/obstructions, and additional congenital pulmonary anomalies were similarly classified. Surgical factors considered were the receipt of ECMO and primary CDH surgical closure (vs. patch or muscle flap). For analyses focused on LOS, factors such as neonatal drug withdrawal, kidney failure, and duration of tube feedings were considered, all three of which were hypothesized to be associated with an increase in LOS among survivors.

### Statistical methods

We tested for associations between each of the variables and the outcomes by univariable analyses. To consider variables that may be independently associated with the selected outcome, factors that (1) varied between centers and (2) were significant to the *p* < 0.2 level in univariable analyses were assessed in the multivariable model and retained by backward selection. Variables that were significant in univariable analyses were omitted in multivariable models if their significance was not retained (*p* < 0.05).

Dichotomous outcomes, such as inpatient survival and the composite of inpatient survival without receiving ECMO were modeled using logistic regression techniques. In these models, gestational age at birth and birthweight were considered separately due to their anticipated collinearity. We report models and adjusted receiver operating characteristic curves (ROC) including ‘center’ as a fixed effect. Goodness-of-fit statistics were computed to determine how well the generated equation fit the observed data and outcomes. Then, model validation was completed using leave-one-out crossover validation. Calibration curves were displayed to evaluate the model’s performance (Supplementary Figs. A,B).

The outcome of LOS was modeled using generalized linear model for gamma-distributed outcome, with log-link. We report the adjusted odds ratios and their 95% confidence interval of the fitted model and focused this analysis only among inpatient survivors. Standardized Deviance residuals between predicted and observed probabilities on LOS were quantified and reported. The Cox proportional hazard model was considered to analyze infants’ timing to discharge, but the assumption of proportionality was violated.

For each outcome, adjusted odds ratios (aOR) are reported graphically for each center. The referent for the centers’ odds ratios was chosen as the center with the median value of the main outcome of interest from the unadjusted, inter-center comparisons. Thus, the referent center varied between each of the three outcomes. Centers with <30 cases during the study period (15 centers) were omitted from the multivariable analyses. Most of the omitted centers began participation in CHND recently and had fewer than 3 years for patient accrual in CHND. Two centers do not care for infants with CDH in their NICUs and their infants were not captured by the CHND.

We validate the model using leave-one-out-cross-validation, and plotted calibration curves, (Supplementary Figs. C, D, E), to assess the agreement between observed and predicted outcomes. Statistical analyses were completed in SAS Enterprise Guide v8.3 (SAS, Cary NC). The significance level was set at *p* < 0.05. Institutional review board oversight was obtained by each participating center in CHND to enter clinical data into the CHND; for secondary analyses, these analyses were considered exempt by the Stanley Manne Research Institute (Chicago, IL: 2009-14982) as investigators did not have direct access to analytic data sets or patient health information.

## Results

### Study cohorts and baseline risk factors and their associations with survival

From 274,158 database records in the CHND, there were 3823 neonates with a diagnosis of CDH identified from 2010 to 2022. The final cohort included 3639 infants after we excluded 121 who were discharged home prior to their NICU admission (and then readmitted), 55 who experienced CDH repair prior to referral to CHNC centers, and 8 patients who had incomplete outcome data upon manual review. Twenty-seven (27) hospitals were represented with a median of 112 cases/hospital (25–75th %ile = 54–152; range = 30–502).

Table 1 presents the baseline characteristics of the cohort and univariate associations with survival. The overall survival rate was 76.5% [ICV range: 55.4, 90.7%, *p* < 0.001]. Preterm birth before 35 weeks’ gestation, female sex, SGA < 10th centile, 5-min Apgar < 3, acidosis, bloodstream infection (BSI), a less recent birth year, antenatal diagnosis, thoracic liver position, and pneumothorax prior to CDH repair, were each associated with inpatient mortality. Neurologic and genetic/chromosomal diagnoses, kidney failure, and complex congenital heart disease were more common in those patients who did not survive. Variation in these covariates are shown in Supplementary Table.

**Table 1:** Baseline case-mix and risk factors.

Patient Level Characteristics	Inpatient Survival	Inpatient Death	OR 95% CI	*p*
*N* (%)	2782	857		-
Median GA (weeks, 25–75th %ile)	38 [37, 39]	37 [36, 39]	1.1 (1.1, 1.2)	<0.001
Female Sex (*N*, %)	1084 (39)	399 (46.6)	0.73 (0.63, 0.85)	<0.001
SGA (*n*, %)	283 (10.2)	167 (19.5)	0.47 (0.38, 0.58)	<0.001
5 min Apgar < 3 (*n*, %)	71 (2.6)	101 (11.8)	0.19 (0.14, 0.26)	<0.001
Any BSI (*n*, %)	226 (8.1)	118 (13.8)	0.55 (0.44, 0.7)	<0.001
Birth year				
2010–2013	600 (21.6)	236 (27.5)	referent	
2014–2016	584 (21)	225 (26.3)	1.02 (0.82, 1.27)	0.85
2017–2019	823 (29.6)	204 (23.8)	1.59 (1.28, 1.97)	<0.001
2020–2023	775 (27.9)	192 (22.4)	1.59 (1.28, 1.97)	<0.001
Antenatal diagnosis (*n*, %)	1846 (66.4)	694 (81)	0.46 (0.38, 0.56)	<0.001
Pre-repair thoracostomy tube (*n*, %)	104 (3.7)	129 (15.1)	0.22 (0.17, 0.29)	<0.001
Either ASD OR VSD or AVCanal (*n*, %)	426 (15.3)	168 (19.6)	0.74 (0.61, 0.9)	0.003
Lowest pH in first 12 h after admission (median, q1,q3)	7.3 [7.2, 7.3]	7.1 [7, 7.3]	modeled as categorical	-
<7.0	132 (4.7)	214 (25)	referent	-
7.0–7.19	581 (20.9)	288 (33.6)	3.27 (2.52, 4.24)	<0.001
7.2–7.29	712 (25.6)	146 (17)	7.9 (5.97, 10.47)	<0.001
7.3–7.45	963 (34.6)	107 (12.5)	14.58 (10.86, 19.59)	<0.001
>7.45+	94 (3.4)	16 (1.9)	9.52 (5.37, 16.88)	<0.001
Liver Position in Thorax (*n*, %)	1271 (45.7)	509 (59.4)	0.58 (0.49, 0.67)	<0.001
Age at referral (days)	1 [1,2]	1 [1,1]	1.2 (1.1, 1.3)	0.008
PCO2 (median, q1,q3)	51 [41.1, 66]	70 [52.8, 95]	modeled as categorical	.
<60 mmHg	1647 (59.2)	278 (32.4)	3.43 (2.89, 4.06)	<0.001
ECMO (*n*, %)	477 (17.1)	473 (55.2)	0.17 (0.14, 0.2)	<0.001
left sided CDH	2107 (75.7)	639 (74.6)	1.06 (0.87, 1.28)	0.567
Primary repair (*n*, %)	1285 (46.2)	63 (7.4)	5.06 (3.82, 6.71)	<0.001
Birthweight, kg (median with 25–75th %ile)	3080 [2700, 3430]	2760 [2270, 3180]	1.1 (1.1, 1.1)	<0.001
Race (*n*, %) NH-White (1)	1544 (55.5)	425 (49.6)	referent	.
NH-Black (2)	292 (10.5)	163 (19)	0.49 (0.4, 0.61)	<0.001
NH-other (3)	241 (8.7)	63 (7.4)	1.05 (0.78, 1.42)	0.734
Hispanic (4)	556 (20)	155 (18.1)	0.99 (0.8, 1.22)	0.905
Comorbidities				
Neurologic Diagnoses	111 (4)	89 (10.4)	0.36 (0.27, 0.48)	<0.001
Complex Cardiac Congenital Malformation	110 (4)	106 (12.4)	0.29 (0.22, 0.39)	<0.001
ASD/VSD	426 (15.3)	166 (19.4)	0.68 (0.55, 0.83)	<0.001
Other CHD	81 (2.9)	92 (10.7)	0.23 (0.17, 0.32)	<0.001
None	2275 (81.8)	599 (69.9)	referent	.
ASD (*n*, %)	274 (9.8)	73 (8.5)	1.17 (0.9, 1.54)	0.247
VSD (*n*, %)	218 (7.8)	118 (13.8)	0.53 (0.42, 0.68)	<0.001
Genetic/Chromosomal Abnormalities	96 (3.5)	74 (8.6)	0.38 (0.28, 0.52)	<0.001
Kidney Failure	67 (2.4)	143 (16.7)	0.12 (0.09, 0.17)	<0.001
Pulmonary Abnormalities	19 (0.7)	17 (2)	0.34 (0.18, 0.66)	0.001
Airway Malacia	117 (4.2)	33 (3.9)	1.1 (0.74, 1.63)	0.648
Gastrointestinal disorders	206 (7.4)	62 (7.2)	1.03 (0.76, 1.38)	0.868

### Inter-center variation in inpatient survival

**Fig. 1:**
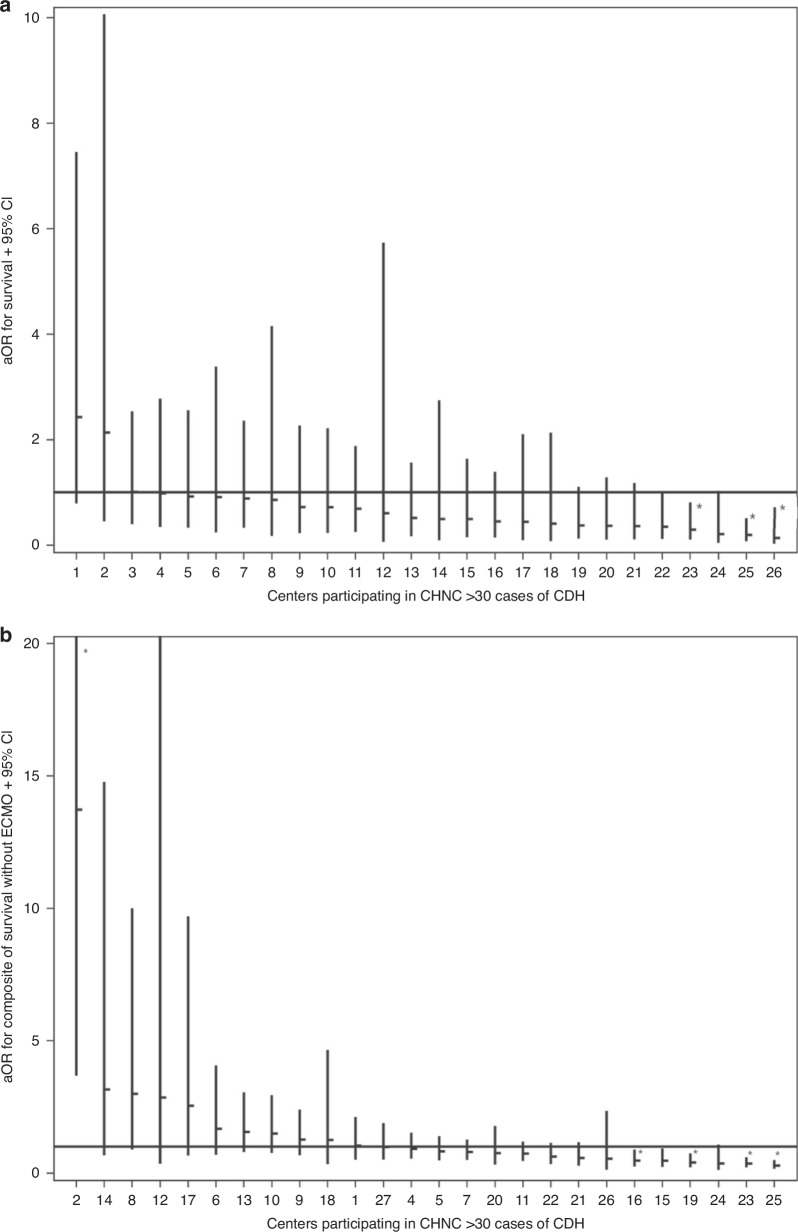
Adjusted survival odds ratios (aOR) by center. **a** This panel illustrates the adjusted odds ratios (aOR) for survival across centers 1–26, including patients treated with and without ECMO. The centers are organized from highest to lowest aOR. Each horizontal dash represents the aOR for a center, with vertical lines indicating the 95% confidence intervals (CI). **b** This panel shows the aOR for survival for each center, similarly, organized from highest to lowest aOR. The aOR for each center is displayed as a horizontal dash, with vertical lines depicting the corresponding 95% CI.

The logistic regression model for survival as an outcome and for estimation of center-specific adjusted survival is summarized in Table 2 and Fig. 1a. The area under the ROC curve was 0.86 (goodness-of-fit, *Χ*^2^ = 14.03, *p* = 0.08). The multivariable model for predicting survival in CDH incorporated several patient-level characteristics; each of these characteristics significantly varied between centers.

**Table 2:** Multivariable model for predicting CDH survival.

Patient Level Characteristics	OR 95% CI	*p*
*N* (%)		
Median GA (weeks, 25–75th %ile)	1.09 (1.02, 1.16)	0.008
Female Sex (*N*, %)	0.67 (0.51, 0.89)	0.006
SGA (*n*, %)	0.65 (0.43, 0.98)	0.039
5 min Apgar < 3 (*n*, %)	0.47 (0.27, 0.83)	0.009
Any BSI (*n*, %)	0.36 (0.25, 0.5)	<0.001
Birth year		
2010–2013	referent	
2014–2016	0.93 (0.63, 1.38)	0.716
2017–2019	1.73 (1.15, 2.62)	0.009
2020–2023	1.52 (1, 2.32)	0.051
Antenatal diagnosis (*n*, %)	0.63 (0.43, 0.94)	0.022
Pre-repair thoracostomy tube (*n*, %)	0.46 (0.29, 0.74)	0.001
Congenital Heart Disease		
None	referent	
ASD/VSD	0.83 (0.58, 1.2)	0.322
Other CHD	0.29 (0.16,0.5)	<0.001
Lowest pH in first 12 h after admission		
<7.0	referent	
7.0–7.19	1.84 (1.19, 2.83)	0.006
7.2–7.29	3.75 (2.33, 6.03)	<0.001
7.3–7.45	4.96 (2.97, 8.28)	<0.001
>7.45+	5.8 (2.1, 16.03)	<0.001
Liver Position in Thorax (*n*, %)	0.5 (0.36, 0.7)	<0.001
Primary repair (*n*, %)	2.36 (1.63, 3.41)	<0.001
Genetic Diagnoses	0.46 (0.23, 0.9)	0.024
Kidney Failure	0.06 (0.04, 0.11)	<0.001

SGA criteria based on Olsen et al.^11^; Each center was included in the models as a fixed effect.

*BSI* blood stream infection, culture positive only, *ASD* atrial septal defect, *VSD* ventricular septal defect, *AVSD* atrioventricular septal defect.

Data from individual centers were included as a fixed effect to account for center-level variations. By dividing the highest center aOR with the lowest center aOR, an 18-fold, risk-adjusted survival range of ICV was observed across participating centers (Fig. 1a). Among 27 centers, 3 were identified as having odds of survival significantly lower than the referent center. None were identified as significantly higher (Fig. 1a).

### Inter-center variation in the composite outcome of Inpatient Survival without ECMO

Table 3 outlines key factors influencing survival without ECMO in infants with CDH. As expected, gestational age < 35 weeks, SGA status, low 5-min Apgar scores, antenatal diagnosis, and BSI were associated with unfavorable outcomes, while, more recent birth years and absence of pre-repair pneumothorax were favorable. Congenital heart defects, especially complex CHD, and lower initial pH levels were linked to either hospital mortality or ECMO. We used logistic regression to predict the composite of survival without receiving ECMO depicted in Table 4 (the area under the ROC curve = 0.86).

**Table 3:** Unadjusted associations for composite outcome of survival without receiving ECMO among infants with CDH.

Variable(s)	Died or Received ECMO	Survived w/o ECMO	OR 95% CI	*p*
*N* (%)	1334	2305		
Gestational age at birth < 35 weeks	168 (12.6)	231 (10)	0.77 (0.63, 0.95)	0.017
Female Sex (*N*, %)	597 (44.8)	886 (38.4)	0.77 (0.67, 0.88)	<0.001
SGA < 10th centile (*n*, %)	212 (15.9)	238 (10.3)	0.61 (0.5, 0.74)	<0.001
5 min Apgar < 3 (*n*, %)	119 (8.9)	53 (2.3)	0.24 (0.17, 0.33)	<0.001
Any BSI (*n*, %)	192 (14.4)	152 (6.6)	0.42(0.34, 0.53)	<0.001
Birth Year				<0.001
2010–2013	366 (27.4)	470 (20.4)	referent	.
2014–2016	327 (24.5)	482 (20.9)	1.15 (0.94, 1.4)	0.168
2017–2019	336 (25.2)	691 (30)	1.6 (1.33, 1.93)	<0.001
2020–2023	305 (22.9)	662 (28.7)	1.69 (1.39, 2.05)	<0.001
Antenatal diagnosis (*n*, %)	1076 (80.7)	1464 (63.5)	0.42 (0.36, 0.49)	<0.001
Pre-repair pneumothorax (*n*, %)	179 (13.4)	54 (2.3)	0.15 (0.11, 0.21)	<0.001
Congenital heart disease (CHD)				
ASD/VSD	245 (18.4)	347 (15.1)	0.73 (0.61, 0.88)	0.001
Other CHD	110 (8.2)	63 (2.7)	0.3 (0.22, 0.41)	<0.001
None	979 (73.4)	1895 (82.2)	referent	-
ASD (*n*, %)	126 (9.4)	221 (9.6)	1.02 (0.81, 1.28)	0.888
VSD (*n*, %)	153 (11.5)	183 (7.9)	0.67 (0.53, 0.83)	<0.001
Either ASD OR VSD or AVSD (*n*, %)	247 (18.5)	347 (15.1)	0.78 (0.65, 0.93)	0.070
Lowest pH in first 12 h after admission	7.1 [7,7.3]	7.3 [7.2,7.4]		-
<7.0	282 (21.1)	64 (2.8)	referent	-
7.0–7.19	467 (35)	402 (17.4)	3.79 (2.8, 5.13)	<0.001
7.2–7.29	269 (20.2)	589 (25.6)	9.65 (7.1, 13.12)	<0.001
7.3–7.45	184 (13.8)	886 (38.4)	21.22 (15.49, 29.05)	<0.001
>7.45+	24 (1.8)	86 (3.7)	15.79 (9.32, 26.76)	<0.001
Thoracic liver position (*n*, %)	862 (64.6)	918 (39.8)	0.36 (0.32, 0.42)	<0.001
Median age at referral (days, 25–75th %ile)	1 [1,1]	1 [1,2]	1.2(1.1, 1.3)	0.001
Median maximum PCO2 in first 12 h after referral (25–75th %ile)	68.3 [52, 90]	49 [40.8,61]		-
PCO2 < 60 mmHg	467 (35)	1455 (63.1)	4.12 (3.54, 4.79)	<0.001
ECMO (*n*, %)	950 (71.2)		0 (0, 3.273474E153)	0.920
Left-sided CDH	985 (73.8)	1761 (76.4)	1.27 (1.07, 1.5)	0.006
Primary CDH repair (*n*, %)	164 (12.3)	1184 (51.4)	5.46 (4.51, 6.61)	<0.001
Median birthweight (g, 25–75th %ile)	2915 [2500, 3270]	3080 [2676, 3440]	1(1,1.1)	<0.001
Race/ethnicity (*n*, %)				
non-Hispanic White	697 (52.2)	1272 (55.2)	referent	<0.001
non-Hispanic Black	233 (17.5)	222 (9.6)	0.52 (0.43, 0.64)	<0.001
non-Hispanic Other	100 (7.5)	204 (8.9)	1.12 (0.86, 1.44)	0.395
Hispanic ethnicity	237 (17.8)	474 (20.6)	1.1 (0.91, 1.31)	0.322
Post-natal drug withdrawal (*n*, %)	219 (16.4)	294 (12.8)	0.74 (0.62, 0.9)	0.002
Neurologic diagnoses (*n*, %)	139 (10.4)	61 (2.6)	0.23 (0.17, 0.32)	<0.001
Genetic/chromosomal diagnoses (*n*, %)	85 (6.4)	85 (3.7)	0.56 (0.41, 0.77)	<0.001
Kidney Failure (*n*, %)	179 (13.4)	31 (1.3)	0.09 (0.06, 0.13)	<0.001
Congenital pulmonary abnormalities (non-CDH), (*n*, %)	22 (1.6)	14 (0.6)	0.36 (0.19, 0.71)	0.003
Airway Malacia (*n*, %)	71 (5.3)	79 (3.4)	0.63 (0.45, 0.88)	0.006
Gastrointestinal Disorders (*n*, %)	102 (7.6)	166 (7.2)	0.94 (0.73, 1.21)	0.618

SGA criteria based on Olsen et al.^11^.

*BSI* blood stream infection, culture positive only, *ASD* atrial septal defect, *VSD* ventricular septal defect, *AVSD* atrioventricular septal defect, *ECMO* extracorporeal support.

**Table 4:** Multivariable analyses for Association on composite outcome of Inpatient Survival without Receiving Extracorporeal Support.

Variable	Odds Ratio (OR)	95% CI		*P*
Gestational age (24–42 weeks) at birth	0.907	0.859	0.958	0.0005
Female Sex	0.76	0.608	0.949	0.0155
SGA < 10th centile	1.116	0.785	1.588	0.5406
5-min Apgar < 3	0.634	0.364	1.103	0.1068
Confirmed BSI	0.449	0.327	0.617	<.0001
Year of Birth (referent 2010–12)				
2014–2016	1.518	1.104	2.086	0.0101
2017–2019	1.943	1.418	2.662	<0.0001
2020–2023	1.879	1.36	2.595	0.0001
Antenatal Diagnosis of CDH	0.742	0.559	0.985	0.039
Pre-repair thoracostomy tube	0.251	0.156	0.403	<0.0001
Congenital Heart Disease				
ASD/VSD	0.834	0.613	1.134	0.2465
Other complex CHD	0.368	0.214	0.634	0.0003
Lowest pH in first 12 h after admission (referent pH < 7.0)				
7.0–7.19	2.411	1.578	3.685	<0.0001
7.20–7.29	5.484	3.539	8.5	<0.0001
7.30–7.45	10.11	6.41	15.945	<0.0001
>7.45	12.944	5.914	28.332	<0.0001
Thoracic liver position	0.363	0.284	0.463	<0.0001
Primary surgical repair	2.779	2.143	3.603	<0.0001
Genetic Diagnosis	0.684	0.364	1.288	0.2396
Neurologic Disorder/Anomaly	0.316	0.191	0.52	<0.0001
Kidney Failure	0.064	0.033	0.125	<0.0001
CHNC Hospital (*n* = 29)	–	–	–	<0.0001

Each center was again included as a fixed effect in the analysis. Center was significantly associated with the composite outcome of inpatient survival without receiving ECMO (overall: 63.3% [ICV range: = 38.6, 87.9%, *p* < 0.001] in unadjusted analysis). This association persisted after including covariates representing patient-level adjustments (Table 4 and Fig. 1b). Each of these covariates also varied between centers.

Four of 27 centers were identified as having significantly lower odds of the composite outcome, whereas 1 of 27 centers was identified as having a significantly higher odds of the composite outcome (Fig. 1b). Comparing the overall odds of survival to survival without ECMO, two centers were identified as significantly higher adjusted odds of these favorable outcomes. Conversely, none of the centers were identified as significantly worse simultaneously in both comparisons. The observed area under the ROC curve was 0.86. In a subgroup analysis restricting the cohort to infants born >34 weeks’ gestation, these results were nearly identical (AUC = 0.87).

A specific comparison of these two main outcomes is necessary. Inpatient survival was observed as 76.5%. Also, the composite outcome of inpatient survival without ECMO was noted to be 63.3%. Thus, the attributable increase in inpatient survival due to ECMO was observed to be 13.2% in this multicenter cohort of infants with CDH.

### Inter-center variation in LOS for surviving infants with CDH

**Fig. 2:**
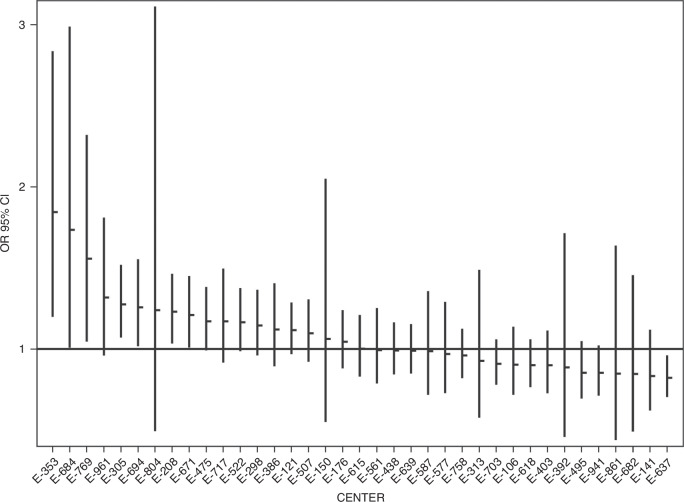
Adjusted hospital length of stay (LOS) for Survivors of Congenital Diaphragmatic Hernia (CDH) by Center. Figure 2 displays the adjusted hospital length of stay (LOS) for CDH survivors across different treatment centers. The X-axis identifies each center, while the Y-axis represents the LOS, adjusted for relevant patient and clinical factors. The data points highlight the variability in LOS among the centers, demonstrating differences in patient outcomes across the institutions included in the study.

The overall median LOS was 50 days [ICV range: 29, 68 days, *p* < 0.001]. Associations between patient factors and LOS among survivors are shown in Table 5. Overall, the adjusted inpatient LOS among survivors varied 3.3-fold between the 27 centers. After risk adjustment, the model predicts that patients at 5 of 27 centers have a longer hospital stay than the referent center while 1 of 27 centers had significantly shorter LOS. (Fig. 2). No center was flagged as falling into the better or worse category for both outcomes, including LOS and survival with or without ECMO. We assessed the fit of this model using residual plots, with standardized deviance residuals between –2 and +2 indicating adequately fitted observations. In our analyses, only 1.82% of residuals were measured to exceed this range.

**Table 5:** Adjusted associations for length of stay.

Variable/Characteristic	ADJUSTED	*p*
*N* (%)	OR 95% CI	
Gestational age at birth <35 weeks	1.59 (1.47, 1.72)	<0.001
SGA < 10th centile (*n*, %)	1.18 (1.09, 1.27)	<0.001
Any BSI (*n*, %)	1.35 (1.25, 1.46)	<0.001
Antenatal diagnosis	1.14 (1.09, 1.19)	<0.001
Congenital heart disease (CHD)		
None	referent	.
ASD/VSD	1.11 (1.04, 1.18)	<0.001
Other CHD	1.29 (1.13, 1.48)	<0.001
pH		
<7	referent	.
7.0–7.19	0.99 (0.89, 1.09)	<0.001
7.2–7.29	0.94 (0.85, 1.04)	<0.001
7.3–7.45	0.85 (0.77, 0.95)	<0.001
>7.45	0.94 (0.81, 1.08)	<0.001
Thoracic Liver Position (*n*, %)	1.3 (1.24, 1.36)	<0.001
ECMO (*n*, %)	1.53 (1.44, 1.62)	<0.001
Primary CDH repair (*n*, %)	0.8 (0.76, 0.83)	<0.001
Race (*n*, %)		
NH-White (1)	referent	.
NH-Black (2)	1.08 (1, 1.16)	<0.001
NH-other (3)	1.08 (1.01, 1.17)	0.039
Hispanic (4)	1.06 (1, 1.11)	0.116
Drug withdrawal, acquired	1.25 (1.18, 1.33)	<0.001
Genetic Diagnoses	1.33 (1.17, 1.52)	<0.001
Airway Malacia	1.62 (1.45, 1.82)	<0.001
Gastrointestinal Disorders	1.35 (1.24, 1.47)	<0.001
Tube feedings at discharge	1.7 (1.62, 1.78)	<0.001

## Discussion

To build on the previous body of research evaluating the role of center and CDH specific outcomes, we undertook this study drawing on the comprehensive data from the CHND. Our study quantified the risk-adjusted ICV of three outcomes: survival, the composite of inpatient survival without receiving ECMO, and length of stay in survivors with CDH. We discovered pronounced inter-center variability in these outcomes, even when accounting for case-mix and other risk factors. In line with improvement science principles, our findings underscore the importance of addressing inter-center variability in CDH outcomes. Identifying significant variations in survival rates, ECMO use, and length of stay is a critical first step to perform future investigations surround medical diagnostic and management strategies, case-load, ECMO experience, and complication prevention. Qualitative queries across centers with disparate outcomes will help inform us which concepts may be beneficial to improve outcomes across hospitals participating in CHNC. These necessary inquiries are planned for to follow-up these findings.

Our findings are consistent with prior studies demonstrating ICV in survival for CDH.^2–5,12,13^ Some of this work came from the CAPSnet registry elucidating volume survival relationships.^5^ The international CDHSG has shown similar variations demonstrated by Jancelewicz et al., who examined the relationship between volume and survival, and the use of ECMO based on risk and center volume.^12,14^ This referenced study demonstrated effect modification: that ECLS in high-risk patients (PCO2 ≥ 60 mmHg) conferred a lower risk of mortality in centers with high volumes of infants with CDH. However, a quantified ICV in inpatient outcomes was not reported.

Perhaps the study most comparable to ours utilized the ELSO registry where centers were compared based on their standardized mortality ratios specific to CDH, and in that study of the 106 eligible centers, 13 centers were reported with significantly lower odds of survival and 7 were reported with significantly higher.^3^ Using the ELSO registry, infants who did not receive ECLS were not examined. Our current analysis identified 3 centers out of 27 as having adjusted odds of increased survival compared to the remaining centers. Although methodologies and cohorts differ, both demonstrate that some centers were identified as having significantly better or worse expected survival compared to others. The current study is novel as it is the first study where the exposure variable is center and population includes neonates from regional, level IV NICUs and is inclusive of affected infants regardless of their ECMO status. Partially given the threshold to include centers with ≥ 30 cases, this study was less focused on volume-outcome relationships which are more established.

The study suggests an attributable increase in inpatient survival due to ECMO (13.2%), while also highlighting the variability in ECMO application across participating centers, with differing subjective indications both between and within these centers. Given the known risks and outcomes,^15^ particularly with the more common veno-arterial approach in infants with CDH, it raises the possibility that some ECMO-treated infants might have survived without this intervention. Interestingly, one center in this study had both significantly higher survival odds for both the outcome of inpatient survival and composite outcome of inpatient survival without ECMO. Unlike previous research, our study uniquely compares the composite rates of inpatient survival without ECMO. This mirrors the broader discourse highlighted in the seminal ‘Tale of Two Cities’ papers, where different treatments yield different outcomes, and it underscores that certain centers excel in achieving high survival with minimal reliance on ECMO.^16,17^

Using the center adjusted equations, we have created models for CHNC participating centers to predict the probability of these outcomes, and anticipated LOS, for infants with CDH. Aside from this study where a large database is used to study CDH-specific LOS and compare centers, Lewit et al. also studied CDH-specific LOS using the administrative dataset, Pediatric Health Information System.^12^ In this study, Lewit et al. identified high-volume centers having longer LOS.^12^ In our study, 5 centers were identified with increased LOS and 1 significantly shorter LOS. Analogous to the Neonatal Research Network’s extremely low birthweight infant calculator to estimate risks of survival and neurodevelopmental consequences, these equations can be used to estimate the probability of survival, the composite outcome of survival without ECMO, and inpatient LOS. We believe these may augment family counseling, allocation of resources, referral practices, and most importantly, local, focused quality improvement activities. Moreover, we propose these models be considered for quality benchmarking standards for reporting.

It may be hypothesized that the observed differences in risk-adjusted LOS may be attributed to unmeasured, or unknown markers illness severity. We submit that this is likely true to a partial degree as the care and management of infants with CDH is subjective with limited evidence on when or how to extubate successfully, how to efficiently feed infants, how to manage pulmonary hypertension, how to discharge complex patients from the NICU, and how to arrange effective, timely, and convenient follow-up for infants and their families. Based on our group’s clinical experiences and practices, it is clear to us that some aspects of illness severity intersect with the quality and breadth of patient and family services provided by NICUs and their hospitals.

Numerous institutions have contributed higher standards of care by developing clinical guidelines and establishing specialized CDH teams.^14^ These guidelines, published by various authoritative bodies in Europe and North America, as well as by the Extracorporeal Life Support Organization (ELSO), reflect a concerted effort to minimize variations in patient management.^18–21^ Despite these efforts, it is noteworthy that the overall mortality rates associated with CDH have remained high, particularly for the group receiving extracorporeal life support (ECMO),^22^ despite some improvement over the past decade described by several groups. Despite success at experienced centers and published protocols, discrepancies in care remain among institutions, suggesting that decisions, including those regarding operative care may be influenced by the unique experiences and capabilities of each center.^23^

This study is subject to several limitations. In the realm of CDH research, among the large datasets available, CHND and ELSO are the primary resources permitted for studies where the treatment center is the exposure variable. All databases have certain limitations. One limitation of CHNC database is that it does not encompass the wider spectrum of ALL institutions in the USA and Canada. While ELSO provides a robust dataset for inquiries specific to ECMO, it naturally does not encompass patients who did not receive ECMO treatment. Similarly, CDHSG data have not yet been released for direct comparison of centers. Consequently, for CDH research that investigates center-based outcomes, including both ECMO-treated and non-ECMO-treated patients, the CHND emerges as the most suitable and inclusive database again where center is the primary research question.

This study does not include infants who died prior to referral or those who were not referred, limiting the generalizability of the findings to all infants with CDH. Additionally, while infants with CDH often have complex medical issues, not all of these were captured, and there may be unmeasured or unknown variables that could confound the associations observed. It is likely that center outcomes are themselves an amalgam of factors, not simply medical care but local health resources and access to care. Antenatal markers of lung volume were not included in these models as detailed above, and indeed many families did not have access to prenatal MRI. Instead, we aimed to identify factors known typically early in the hospital course and estimate their associations on the chosen outcomes. The large datasets utilized are also prone to potential misclassification of exposures, covariates, or outcomes. Nevertheless, our findings align with those from other registries and studies targeting similar populations, and previous work^3,4,24–27^ has shown limited errors in data entry for the CHNC.

These results suggest multiple conclusions. First, center serves as a proxy for how management and the resultant outcomes differ. This study incorporates adjustments for illness severity of both patient-and center-level data, perhaps narrowing the gap between these important contributors to outcomes. Our results can guide future qualitative and quantitative work to understand efforts adopted by high-performing centers. Such research may elucidate generalizable practices that can be spread, adopted, measured, and disseminated to improve outcomes globally. Second, our reported statistical models could be used by centers to quantify risk factors for our three studied outcomes in individual patients that are based on a set of clinical and demographic characteristics. Many of the characteristics are known soon after birth and thus can be used for early post-natal counseling and anticipatory guidance. Third, in the future therapeutic interventions can be added to these models, and if a specific therapy is related to improved outcomes, these approaches may serve as candidates for rigorous testing in clinical trials. Lastly, we propose these risk-models can become a viable tool for center-based quality metrics to compare, evaluate, and improve future clinical outcomes for infants with CDH in collaborative quality improvement initiatives.

## Supplementary information

Supplementary Figure

Supplementary Figure

Supplementary Table

## Data Availability

The data utilized in this study are sourced from the Children’s Hospital Neonatal Consortium (CHNC) database. Access to individual hospital data is restricted to CHNC member institutions and is governed by CHNC policies, which allow members to access their own data. Compiled, multi-center data are available exclusively to CHNC research staff in accordance with CHNC bylaws. Researchers interested in accessing CHNC data for collaborative studies may do so through formal agreements, subject to CHNC review and approval. For further information, inquiries can be directed to the CHNC Research Committee.
