# Supplementary material for: Congenital diaphragmatic hernia outcomes: navigating center-to-center variability in level 4 NICUs in the Children’s Hospitals Neonatal Consortium
Source: Pediatr Res. 2025 Feb 25;99(3):999–1008. doi: 10.1038/s41390-025-03829-0 (PMC13021515; doi:10.1038/s41390-025-03829-0)
Supplement: Supplementary file 1 — Supplementary Figure [file 41390_2025_3829_MOESM1_ESM.pdf]

Supplemental Figure C

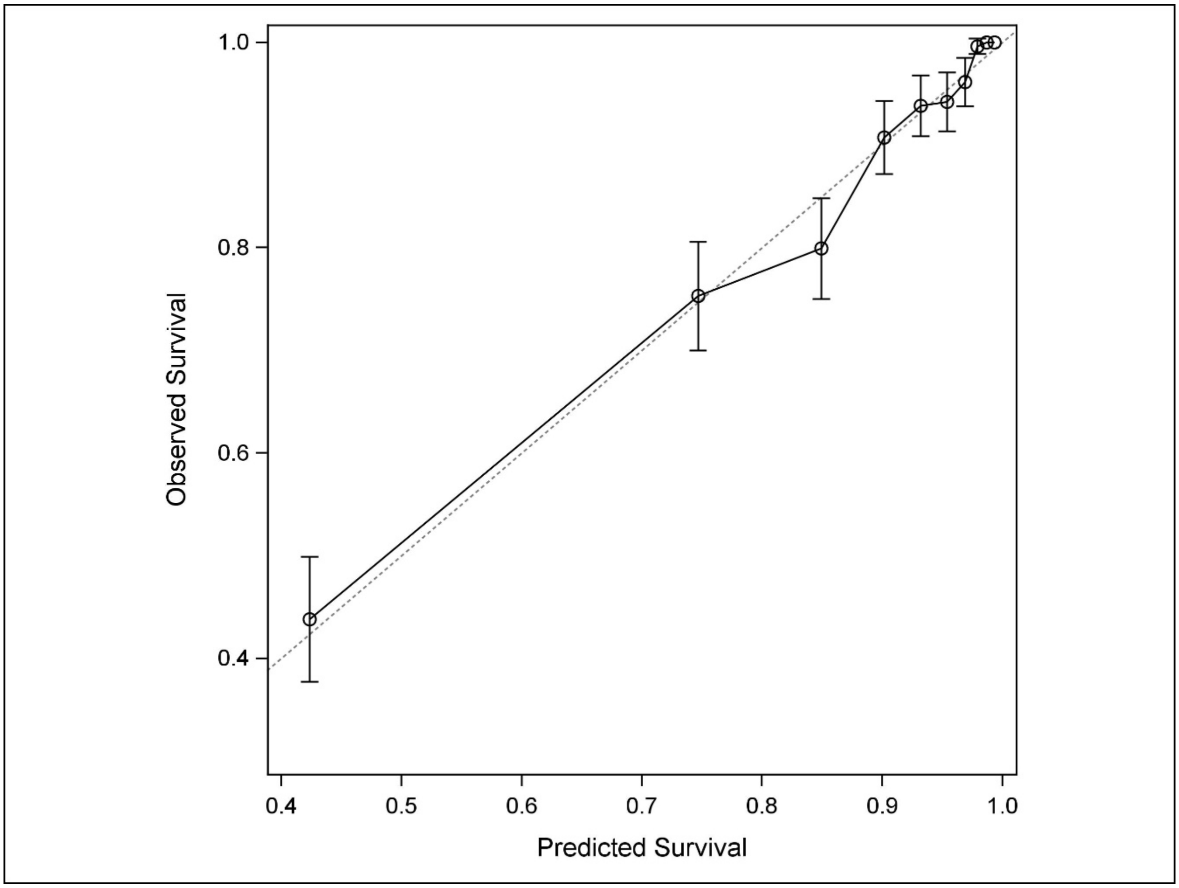

Calibration plot for Observed vs. Expected probability of Inpatient Survival

Supplemental Figure D

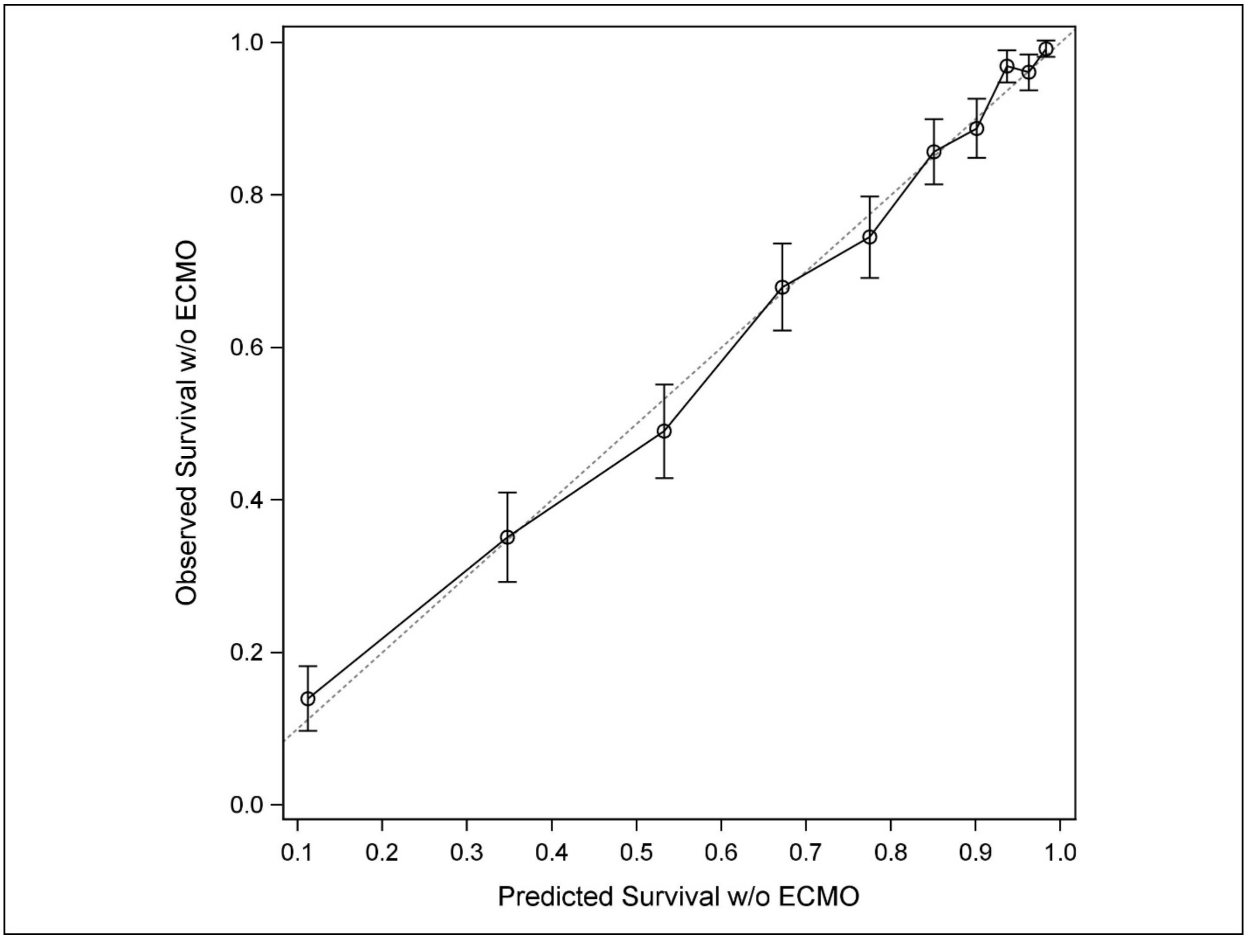

Calibration plot for Observed vs. Expected probability of the Composite Inpatient Survival without ECMO usage

Supplemental Figure E

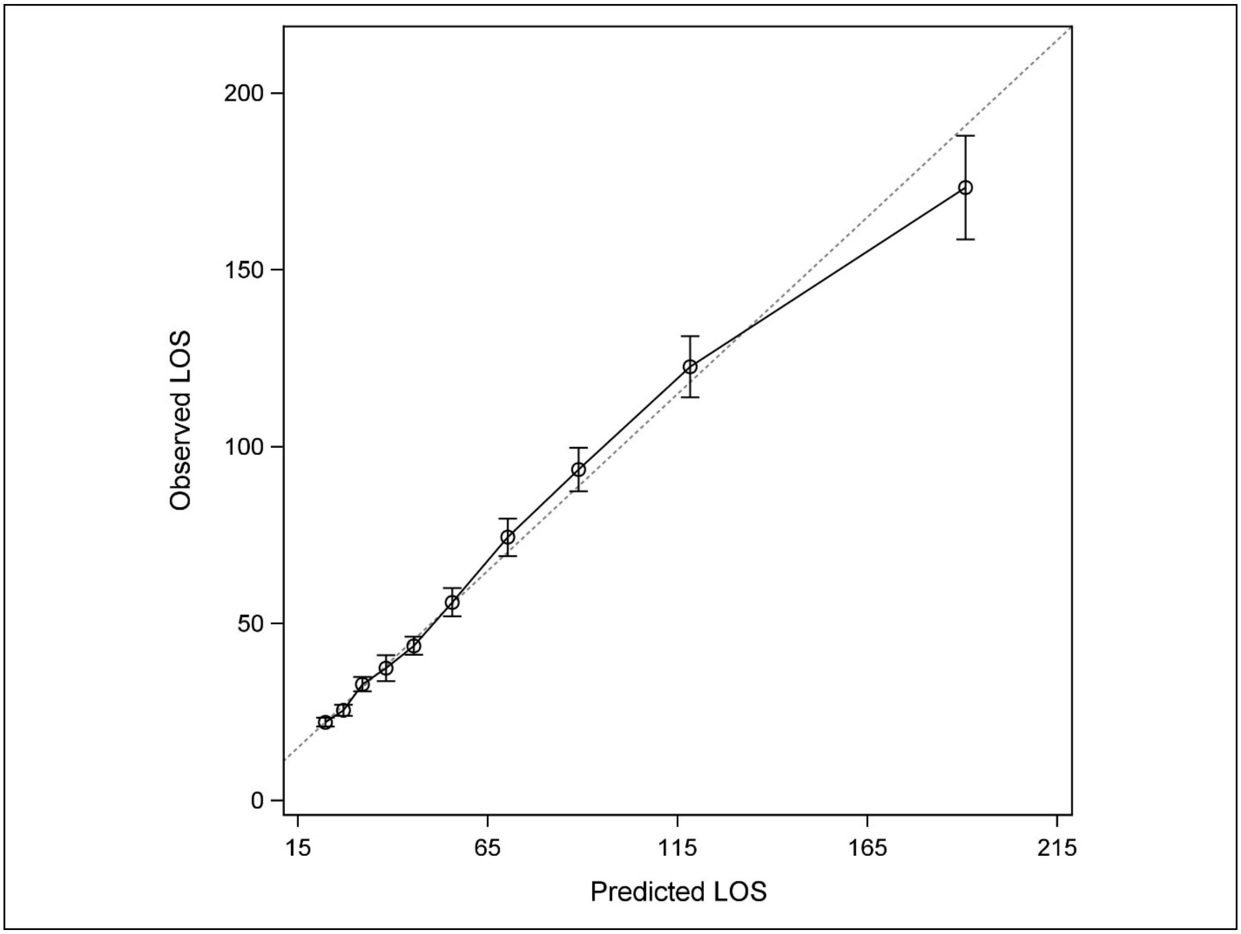

Calibration plot for Observed vs. Expected LOS (days) of Hospital length of Stay Among Survivors with CDH
