# Supplementary material for: Congenital diaphragmatic hernia outcomes: navigating center-to-center variability in level 4 NICUs in the Children’s Hospitals Neonatal Consortium
Source: Pediatr Res. 2025 Feb 25;99(3):999–1008. doi: 10.1038/s41390-025-03829-0 (PMC13021515; doi:10.1038/s41390-025-03829-0)
Supplement: Supplementary file 2 — Supplementary Figure [file 41390_2025_3829_MOESM2_ESM.pdf]

Supplemental Figure A

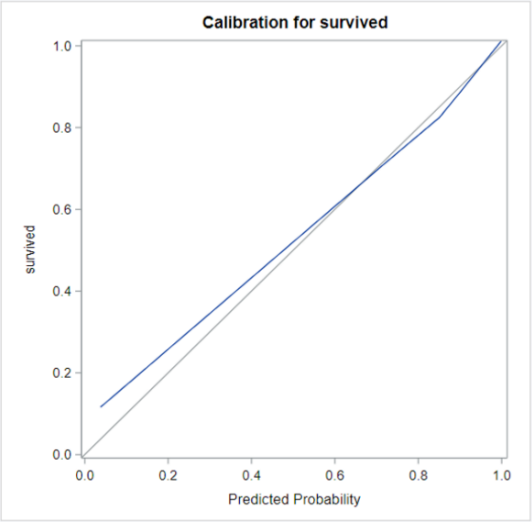

Supplemental Figure B

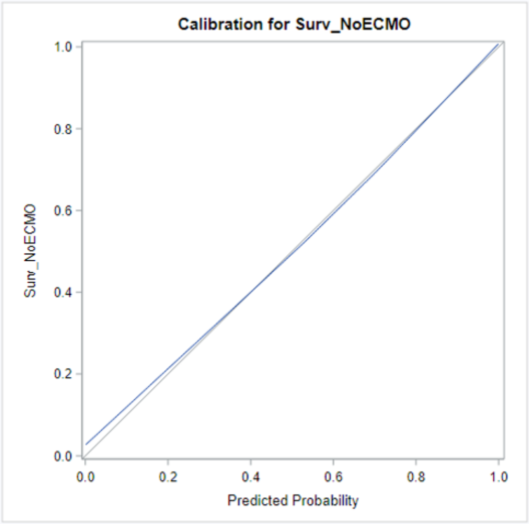

Figure: Observed vs. Expected Calibration Plot for (A) survival and (B) composite of Survival without Receipt of ECMO. Note the high correlation between observed vs. expected probabilities for both outcomes.
