# Supplementary material for: Congenital diaphragmatic hernia outcomes: navigating center-to-center variability in level 4 NICUs in the Children’s Hospitals Neonatal Consortium
Source: Pediatr Res. 2025 Feb 25;99(3):999–1008. doi: 10.1038/s41390-025-03829-0 (PMC13021515; doi:10.1038/s41390-025-03829-0)
Supplement: Supplementary file 3 — Supplementary Table [file 41390_2025_3829_MOESM3_ESM.pdf]

Supplemental Table: Inter-center Variation in selected covariates used in regression models for the three main outcomes

Variable	Median of center values*	25th-75th%ile	Range	P^
Gestational age (weeks)	38	38-38	37-39	<0.001
Age of admission (days)	1	1-1	1-10	<0.001
Lowest pH in the first 12 hours after admission	7.25	7.22-7.28	7.15-7.32	<0.001
ECMO (%)	27.09	12.62-33.86	1.01-50	<0.001
Male sex (%)	58.98	56.14-61.8	48.84-69.23	0.535
Small for gestational age < 10th centile (%)	11.52	9.47-15.25	3.45-30.77	0.006
Culture positive bloodstream infection (%)	8.82	6.36-10.65	3.23-16.28	0.014
5-min Apgar < 3 (%)	4.83	3.34-6.36	1.39-11.11	0.062
Antenatal diagnosis (%)	64.01	58.93-75.52	29.41-88.84	<0.001
ASD/VSD (%)	16.1	12.24-20.52	3.88-38.46	<0.001
Pre-repair pneumothorax (%)	6.45	4.22-8.93	1.39-11.82	0.163
Thoracic liver position (%)	46.6	39.84-52.73	25.64-70.67	<0.001
Genetic diagnosis established (%)	4.58	3.03-5.88	0.97-12.31	0.094
Kidney failure/dysfunction (%)	3.74	2.33-7.97	0.79-37.5	<0.001
Neurologic co-morbidities (multiple diagnoses) (%)	5.83	3.54-8.1	0.79-16.07	0.001
Primary surgical repair (%)	45.35	40.91-59.26	16.57-96	<0.001
Gastrointestinal co-morbidities (multiple diagnoses) (%)	6.47	4.8-8.88	2.73-19.42	0.004
Airway malacia (%)	4.01	2.02-5.42	0.79-12.7	0.001

*The center value is the center percentage for categorical variables, and the center median for continuous variable

^Unadjusted ICV assessed with Wald Chi-Square for categorical variables, Kruskal-Wallis Test for continuous distributions
